# Supplementary material for: IGF2 May Enhance Placental Fatty Acid Metabolism by Regulating Expression of Fatty Acid Carriers in the Growth of Fetus and Placenta during Late Pregnancy in Pigs
Source: Genes (Basel). 2023 Apr 5;14(4):872. doi: 10.3390/genes14040872 (PMC10137774; doi:10.3390/genes14040872)
Supplement: Supplementary file 1 [file genes-14-00872-s001.zip › genes-2228882-supplementary.pdf]

Table S1. Primer information of Bisulfite -PCR

Gene Loci	Primer Sequences (5′ to 3′)	Product Size (bp)	Annealing temp (°C)	References
IGF2-DMR2	OF:<br>OR:	268	55	(Wei *et al.* 2010)
	IF: GGTAGTATTTGAAGTTTAAGAG<br>IR: CTATAAAACTTCCAAACAAACC		55	
H19-CTCF3	OF:<br>OR:	205	50	
	IF:<br>IR:		55	

OF and OR represent external upstream and downstream primers, while IF and IR represent internal upstream and downstream primers, respectively.

Table S2. Body weight of pig fetuses derive from D40, D65, D95 and term pregnancies.

Group	No. of Pregnant Sows	No. of Collected Fetuses	Average Body Weight of Fetuses (g)#
D40	4	45	12.3 ± 1.7
D65	4	41	212.6 ± 29.5
D95	5	49	686.7 ± 164.2
Term	5	45	1393.5 ± 349.3

#Data are expressed as mean ± SD
